# A reported death case of a novel bunyavirus in Shanghai, China

**DOI:** 10.1186/1743-422X-10-187

**Published:** 2013-06-07

**Authors:** Hao Pan, Jiayu Hu, Shelan Liu, Hong Shen, Yiyi Zhu, Jiabing Wu, Xi Zhang, Xin Zhou, Chengmin Wang, Jing Qu, Zheng’an Yuan

**Affiliations:** 1 Shanghai Municipal Center for Disease Control and Prevention, No 1380, West Zhongshan Road, Shanghai, 200336, China; 2 Zhejiang Provincial Center for Disease Control and Prevention, No 630, XinCheng Road, Binjiang District, Hangzhou, Zhejiang Province, 310051, China; 3 Xuhui District Center for Disease Control and Prevention, Xuhui, No 50, Yongchuan Road, Shanghai, 200237, China; 4 Anhui Provincial Center for Disease Control and Prevention, No 12560, Fanhua Road, Hefei, Anhui Province, 230601, China; 5 Institute of Zoology, Chinese Academy of Sciences, No 1, Beichen west Road, Chaoyang District, Beijing, PR, 100000, China; 6 National Institute for Viral Disease Control and Prevention, No 155, Changbai Road, Changping District, Beijing, 102206, PR China

**Keywords:** Severe fever and thrombocytopenia syndrome, Bunyavirus, Genetic analysis

## Abstract

This paper describes the first case of infection with a recently described novel bunyavirus, severe fever with thrombocytopenia syndrome virus (SFTSV), in Shanghai, China. The case is originally from Chizhou City, Anhui province within an endemic area for SFTSV. We describe the etiology, epidemiological characteristics, clinical diagnosis and treatment of this fatal case. This case is unique because major cause of death was renal failure, whereas other reported cases have been due to hemorrhage. The investigation and response to this case provides meaningful insight for the early and rapid diagnosis, treatment, prevention and control of severe fever with thrombocytopenia syndrome virus in non-endemic regions in China and globally.

## Background

A novel member of phlebovirus genus of the *Bunyaviridae* family, severe fever with thrombocytopenia syndrome virus (SFTSV), was first identified in China in 2010 [1,2], Surveillance data indicate that eastern, central, and northeast China are the major endemic areas of the virus, although there have been sporadic cases in non-endemic areas. As suggested by the clinical syndrome it produces (severe fever with thrombocytopenia syndrome, SFTS), the key features of this viral infection are a severe systemic illness characterized by fever and thrombocytopenia.

SFTS mainly occurs in the spring and summer, especially among middle-aged residents who live in mountainous regions [3]. SFTSV infection results in fever, thrombocytopenia, gastrointestinal symptoms, and leukocytopenia [4]. The initial fatality rate for SFTS was 30%, however with improved diagnosis or treatment of the disease, the fatality rate in China has decreased by 10-15% according to 2011 surveillance data. These fatalities were characterized by patients dying from hemorrhage or multiorgan failure [5].

According to the Chinese SFTS diagnosis and treatment guidelines, the case definition for SFTS includes a temperature above 38°C associated with thrombocytopenia, and fever with one or more bleeding symptoms (melena, bleeding gums, skin petechial or ecchymose, conjunctival hyperemia or hemorrhage symptoms). The case is confirmed by virus isolation, the detection of SFTS bunyavirus RNA, by novel bunyavirus IgG antibody seroconversion, and/or by a convalescent titer more than four times higher than in the acute phase [1].

In our analysis, we present the case in terms of infection, diagnosis and treatment and then discuss the epidemiology and virology.

## Case presentation

The case under consideration in this article is a 40 year-old female farmer who lived in the countryside near the hilly areas outside Chizhou city, in Anhui Province, China, which is one of the central endemic areas. The patient had given birth by caesarean section in 1999, and raised her 13 year old in her hometown, while her husband left for work in another city. She had no other basic conditions or infectious diseases; except for a history of shistosomiasis in 2008. There was no history of previous drug or food allergies or blood transfusions. She was admitted to a hospital in Shanghai (400 km/78 miles from Chizhou) in the middle of May 2012.

### Clinical history

The patient had a sudden onset of fever (reaching a peak in temperature of 40°C) accompanied by muscle pain on May 16th 2012. She was reviewed at the local hospital, admitted and commenced on intravenous antibiotics, but no clinical improvement was observed. On May 19th, she was transferred to the hospital in Chizhou where she was treated with aztreonam and ribavirin, but the high fever continued. A tick-borne disease was suspected. On May 21st, the patient was transferred to a hospital in Shanghai, reporting fever, chills, body aches, and diarrhea occurring five to six times a day. The patient was clinically suspected of having SFTSV infection, admitted to the ICU (Intensive Care Unit), was given cefotiam, levodvopropizine, and other supporting and symptomatic treatments, but did not improve. The patient became apathetic on May 22nd and she was given vancomycin and other input platelet therapy, but her condition further deteriorated.

The following day she had a stiff neck, accompanied by enlarged lymph nodes in the neck, axillary and mediastinal regions, and abnormal brain waves on electroencephalogram. On May 25th, the patient was in critical condition with shortness of breath, hypotension to 88/50 mmHg, significant oliguria with acute renal failure, severe acidosis, an abnormal flow index of the liver and kidneys, although there was no skin bleeding or blood stasis. Thereafter, she was treated with prednisone, platelet and plasma transfusions, and hemodialysis, but died on the same day (May 25th) of died of multi-organ failure, including kidney and respiratory failure, disseminated intravascular coagulation and septic shock.

### Basic laboratory investigations

Laboratory tests showed that the patient’s white cell count decreased through most of the course of her illness, but then increased on the day of her death (Table 1). The platelet counts persistently decreased along with thrombin times, and activated partial thromboplastin times (APTTs) were prolonged. Multiorgan dysfunction (including liver and kidney failure) was evidenced by elevated blood urea nitrogen (BUN) and hepatic transaminases (alanine aminotransferase, ALT; aspartate aminotransferase; AST), lactate dehydrogenase (LDH), and creatine kinase (CK). The presence of microscopic hematuria and proteinuria were also documented. These clinical findings are summarized in Table 1.

**Table 1:** Laboratory findings of a confirmed case of infection with SFTSV in Shanghai, China, 2012

**Laboratory variables**	**D4 (5.19)**	**D5 (5.20 9:50 AM)**	**D5 (5.20 12:00 AM)**	**D6 (5.21)**	**D7 (5.22)**	**D8 (5.23)**	**D9 (5.24)**	**D10 (5.25) Death**
WBC (×10^9^/L)	1.32	0.83	0.92	1.19	/	1.20	/	11.57
NEUT (×10^9^/L)	0.94	0.57	0.69	0.7	/	0.8	/	
LYMPH (×10^9^/L)	0.30	0.23	0.20	0.4	/	0.4	/	
PLT (×10^9^/L)	51	40	39	35	/	27	/	61
Red cell count (×10^12^/L)	3.03	3.18	3.09	3.33	/	3.34	/	3.8
Hb(g/L)	97	101	95	105	/	105	/	119
ALT(U/L)	23	/	/	72	/	/	/	254
AST(U/L)	62	/	/	277	/	/	/	2321
LDH(U/L)	320	/	/	1411	/	/	/	
CK(U/L)	154	/	/	495	/	/	/	1180
CK-MB(U/L)	17	/	/	/	/	/	/	196
CK-MM(U/L)	/	/	/	/	/	/	/	984
CR(μ mol/L)	47.2	50.1	/	61	/	/	/	275
BUN(mmol/L)	5.71	3.70	/	3.2	/	/	/	15.9
TT(Second)	/	/	13	/	24.5	/	/	>150
PT(Second)	/	/	13	/	13.4	/	/	23.4
APTT(Second)	/	/	35.50	/	53.7	/	/	140
Positive urine protein	/	/	/	++++	+++	/	/	

*D* day of illness, *WBC* White blood cell, *LYMPH* Lymphocyte, *NEUT* neutrophil, *PLT* platelet, *HGB* hemoglobin, *ALT* alanme aminotransfcrase, *AST* aspartate amino-transferase, *APTT* activated partial thromboplastin time, *TT* thrombm time, *LDH* lactate dehydrogenase, *CK* creatine kinase, *BUN* blood urea nitrogen, *CR* creatinine.

On May 25th the results of an arterial blood gas was consistent with severe metabolic acidosis with respiratory compensation, as follows:

pH: 7.17; PCO_2_: 14.0 mmHg; PO_2_: 110 mmHg; HCO_3_^-^: 5.10 mmol/L; CO_2_-CT: 5.5 mmol/L; BE-ecf: -23.4 mmol/L; SBC: 8.5; BE-b: -21.1 mmol/L; SO_2_: 97%.

### Epidemiology

The patient had several environmental exposures that may have contributed to her acquisition of SFTSV. She was engaged in collecting cotton, rice and tea until two weeks before the onset, and also had a contact history with birds and rodents and other wild animals in the local area. She had no skin damage or history of tick bites. She had had no known exposure to a person with a similar illness in the countryside, and no similar cases were found in the local town where the patient lived. Nineteen people, including three family members and sixteen medical staff (eleven doctors and five nurses) came into contact with the patient during the course of her hospitalization. No secondary cases of SFTS were found in any of these contacts.

### Virology and associated molecular investigations

**Figure 1:**
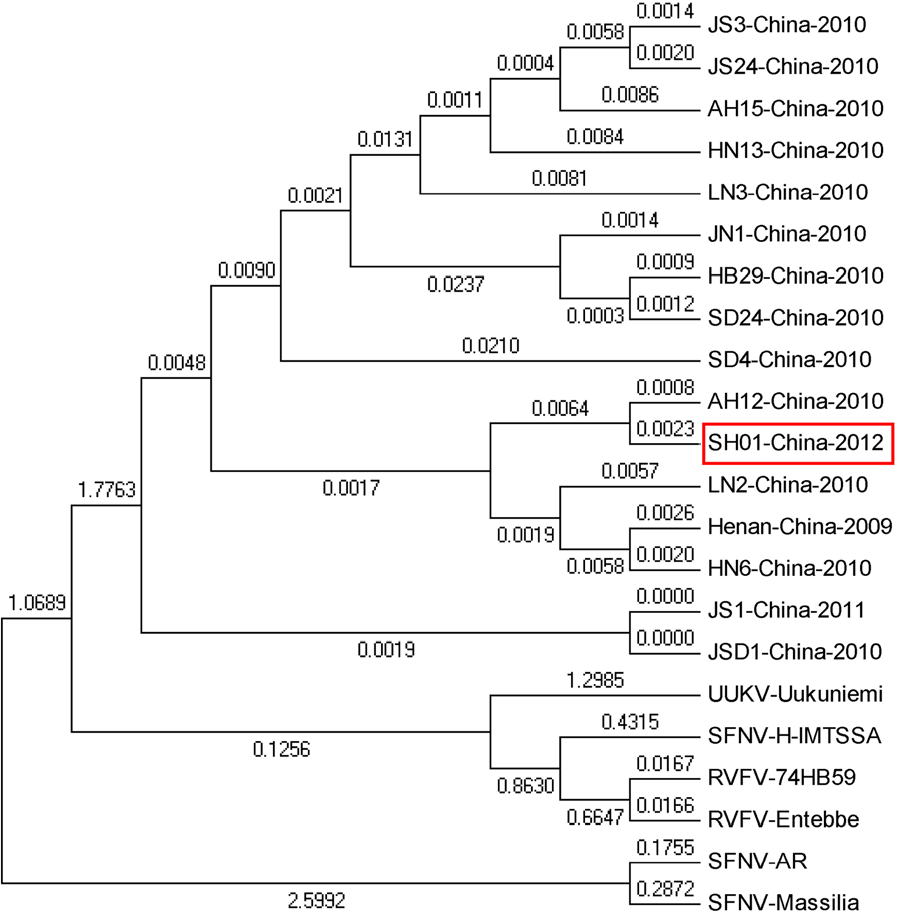
**L gene phylogenetic analysis comparing SFTS bunyavirus strain from Shanghai and other areas, and other phlebovirus species downloaded from GenBank.** RVFV (Valley fever virus); SFNV (Sandfly fever Naples virus); and UUKV (Uukuniemi virus).

On May 25th, blood samples collected on the day of death were sent to Shanghai Municipal Center for Disease Control and Prevention, and confirmed as SFTSV. Confirmation was by real-time reverse transcriptase quantitative PCR [6]. L, S and M segments were sequenced and analyzed by Mega 5.05. The results showed that the L sequence of the patient was closely related (96.3–98.7% nucleotide sequence homology) to the L segment of SFTSV isolated from other areas in China, including Jiangsu, Anhui, Shandong and Henan. In contrast, the L segment only had a 48.7% sequence homology to Rift Valley fever virus (RVFV), 37.2% to Toscann virus, and 50.4% to Uukuniemi virus (UUKV). The phylogenetic tree of nuclear acid sequences of the RNA-dependent RNA polymerase was derived from the L-segment sequence of previously reported SFTSV strains and that from our patient. This shows that our patient’s strain and that of other Chinese SFTSV isolates belong to a single genetic lineage, whereas these are all nearly equidistant from the Sandfly fever and Uukuniemi groups, as previously reported. This result suggests that SFTSV from the patient has one genetic lineage and belongs to a novel group in the phlebovirus genus (see Figure 1).

**Figure 2:**
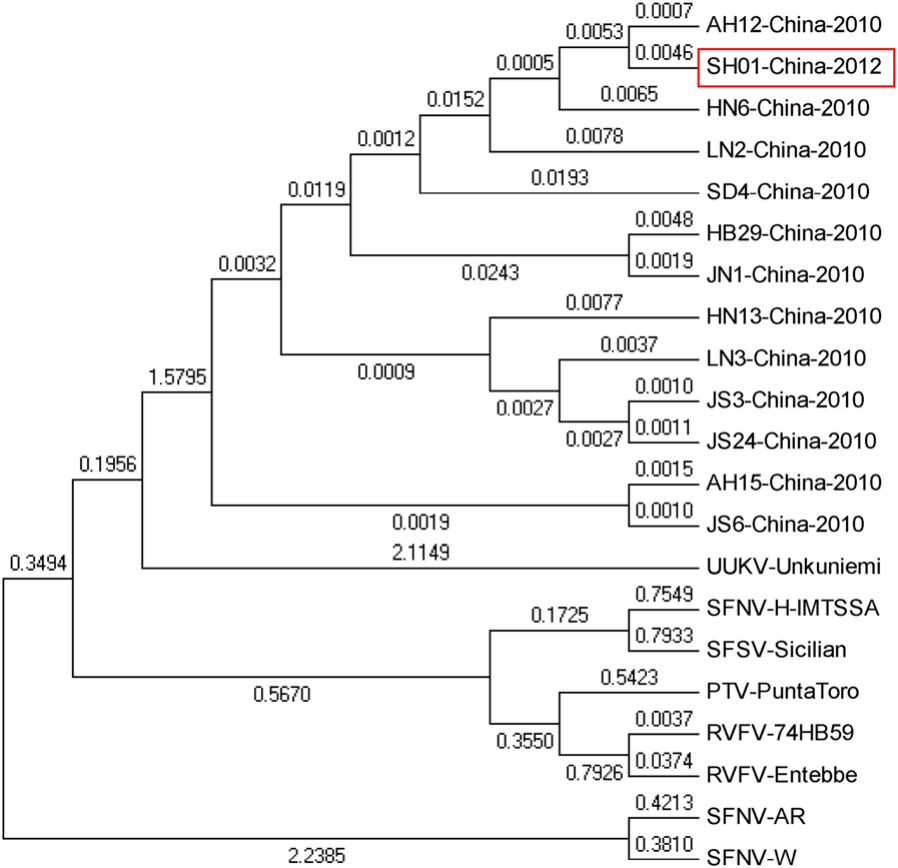
**M gene phylogenetic analysis comparing SFTS bunyavirus strains from Shanghai and other areas, and other phlebovirus species downloaded from GenBank.** RVFV (Valley fever virus); SFNV (Sand fly fever Naples virus); UUKV (Uukuniemi virus); SFSV (Sandfly fever Sicilian virus); and PTV (Punta Toro virus).

The homology of the M sequence in this patient was 97.9–99.4% to other SFTSV strains, but showed only 68.2–68.6% similarity to other phleboviruses. The phylogenetic tree indicated that all Chinese SFTSV M genes are clustered together, but are very distantly related to other phlebovirus groups, such as the Sandfly fever group, (Rift Valley fever virus, Punta Toro virus, Toscana virus, Massila virus, and Sandfly fever Sicilian virus), and the Uukuniemi group (see Figure 2).

**Figure 3:**
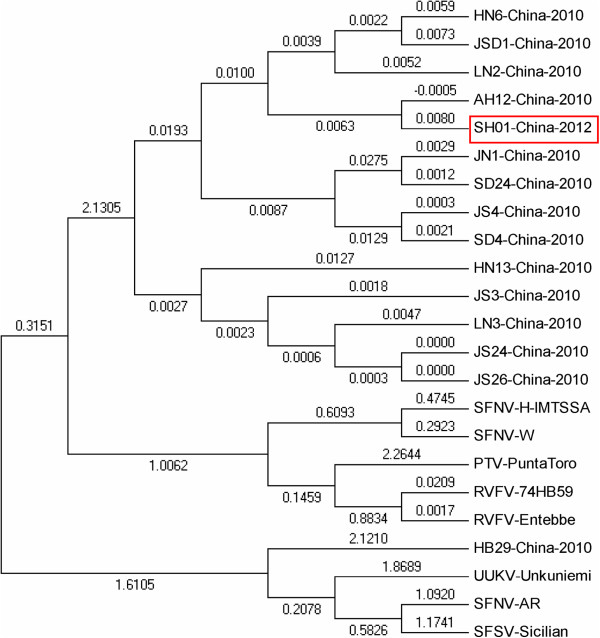
**S gene phylogenetic analysis comparing SFTS bunyavirus strains from Shanghai and other areas, and other phlebovirus species downloaded from GenBank.** RVFV (Valley fever virus); SFNV (Sandfly fever Naples virus); UUKV (Uukuniemi virus); SFSV (Sandfly fever Sicilian virus); and PTV (Punta Toro virus).

The S segment of the patient had 95.0–99.5% homology to other SFTSV strains, but only 32.0–40.2% similarity to other phleboviruses. The phylogenetic tree was very similar to the M and L gene analyses (see Figure 3).

There was no difference in the L, S and M segment mutations between SFTSV strains from the patient who died and previously reported patients with mild disease.

The study was approved by the Ethical Committee of the Shanghai Municipal Center for Disease Control and Prevention.

## Conclusions

The clinical symptoms of SFTS are considered to resemble those of human anaplasmosis, hemorrhagic fever with renal syndrome, and leptospirosis [2,7-10]. This patient presented with fever, gastrointestinal symptoms, leucocytopenia and thrombocytopenia. The potential pathogens causing this clinical presentation can be differentiated with the use of basic and specialized laboratory testing, including microscopy, nucleic acid detection and sequencing, antigen detection, virus isolation, and serum antibody testing. The method of virus acquisition remains to be clearly elucidated. Ticks may serve as one of the candidate vectors of SFTSV, since the RNA has been detected in *Haemaphysalis longicornis* and other tick species [2,11,12]. SFTS, an emerging infectious disease, is progressive and potentially fatal. The epidemiology, pathogenesis and route of transmission of this novel disease are still unclear.

Our patient was a 40 year-old female farmer, who had lived in the hilly areas of Anhui Province, and had no contact with individuals with similar clinical manifestations. Although she had no history of tick bites, it is likely she had contact in the fields while harvesting tea and hay. Further research is required to determine whether ticks, mosquitos, and/or other rodent animals are the vectors and host species of SFTSV.

Epidemiologic investigation indicated that no contacts or other villagers were ill with a similar illness. This suggests that person-to-person transmission was limited, although there are reports in other areas of China that suggested person-to-person transmission [13-15].

The illness duration prior to death in our patient was ten days, but she was confirmed as a SFTSV infection only on the day of her death. The laboratory tests showed that her white blood cell (WBC) count decreased during the early stages, but increased on the day of her death; platelet counts decreased progressively during the early stages and were persistently low until the day of her death. The platelet count decreased to 27× 10^9^/L on the 8^th^ day after the onset of the disease, accompanied with coagulopathy (TT, PT and APTT prolonged), and remained low at 61× 10^9^/L on the day of death despite platelet transfusions. The elevated WBC count on the day of death may reflect a systemic inflammatory response or possible secondary bacterial infection. Her hemoglobin (Hb) and red blood cell (RBC) counts remained stable throughout her illness, consistent with the absence of any hemorrhagic complications from her thrombocytopenia and coagulation disorder. Late in the disease SFTSV can invade the heart, liver, kidney, gastrointestinal tract, and other vital organs, as well as the bloodstream [16]; our case was not tested for focal organ involvement. In the case reported here, kidney and liver dysfunction became more evident, as indicated by the elevated BUN, creatinine and liver transaminases. Severe renal failure caused persistent oliguria, sodium and water retention, resulting in respiratory failure and severe acidosis, and electrolyte disorders. The acute renal failure was considered the major cause of death. This differentiates this case from other reports of SFTSV infection, where the major cause of death was hemorrhage [6,10,17,18].

Molecular analysis confirmed that this patient’s SFTSV had very high homology to previously described strains from other regions in China. Further analysis of viral sequence variation did not reveal any characteristic genetic changes in our patient’s fatal strain when compared to genomes of SFTSV isolated from mild cases. This suggests there are not viral genetic factors affecting variations in virulence and pathogenicity between the fatal and mild cases.

SFTS was previously reported in seven provinces of China: Henan, Hubei, Shandong, Anhui, Jiangsu, Liaoning, and Zhejiang. A national surveillance system for SFTS was built in these regions and nearby non-epidemic areas. Here we report a case of death from SFTSV, in a patient who lived in the Anhui mountain areas (endemic for SFTSV), but was treated and diagnosed in Shanghai. This SFTSV case highlights the potential risk to the healthy population in non-endemic regions, because the patient went to a large city, Shanghai, for medical treatment, and person-to-person transmission has been described [15]. It will be a great challenge for the early diagnosis of SFTS to be carried out in hospitals in non-endemic areas. Earlier diagnosis of SFTSV infection will facilitate prompt institution medical intervention earlier in the course of illness and will hopefully improve outcomes for patients with SFTSV infection. In the future, attention should be put on continuing to strengthen SFTS surveillance and medical staff training, as well as further standardization and improvement of levels of medical treatment for SFTS, along with exploration of potential antiviral therapies.

### Consent

Written informed consent was obtained from the patient for publication of this case report and any accompanying images.

## Abbreviations

PCO2: Partial pressure of arterial carbon dioxygen; PO2: Partial pressure of arterial oxygen; HCO3-: Calculated bicarbonate concentration; CO2-CT: Total carbon dioxide Content; BE-ecf: Base excess extracellular fluid; SBC: Standard bicarbonate concentration; BE-b: Base excess of blood; SO2: Arterial oxygen saturation.

## Competing interests

The authors declare that they have no competing interests.

## Authors’ contributions

HP carried out the epidemiology survey and analysis and drafted the manuscript. JH and SL collected clinical data and conducted statistical analysis of the data. HS, YZ and JW participated in determination of survey on contacts and collected samples. XZ XZ and JQ performed the lab analysis. CW was responsible for the sequence analysis. ZY conceived the study and participated in its design and coordination and helped to draft the manuscript. All authors read and approved the final manuscript.
